# The relationship between living in urban and rural areas of Scotland and children’s physical activity and sedentary levels: a country-wide cross-sectional analysis

**DOI:** 10.1186/s12889-020-8311-y

**Published:** 2020-03-06

**Authors:** Paul McCrorie, Rich Mitchell, Laura Macdonald, Andrew Jones, Emma Coombes, Jasper Schipperijn, Anne Ellaway

**Affiliations:** 1 MRC/CSO Social and Public Health Sciences Unit, University of Glasgow, Glasgow, Scotland; 2 Norwich Medical School, University of East Anglia, Norwich, UK; 3 Department of Sports Science and Clinical Biomechanics, University of Southern Denmark, Odense, Denmark

**Keywords:** Physical activity, Children and young people, Season, Income, Urban and rural

## Abstract

**Background:**

Living in urban or rural environments may influence children’s levels of physical activity and sedentary behaviours. We know little about variations in device-measured physical activity and sedentary levels of urban and rural children using nationally representative samples, or if these differences are moderated by socioeconomic factors or seasonal variation. Moreover, little is known about the influence of ‘walkability’ in the UK context. A greater understanding of these can better inform intervention strategies or policy initiatives at the population level.

**Methods:**

Country-wide cross-sectional study in Scotland in which 774 children (427 girls, 357 boys), aged 10/11 years, wore an accelerometer on one occasion for at least four weekdays and one weekend day. Mean total physical activity, time spent in sedentary, light, and moderate-to-vigorous physical activity (MVPA), per day were extracted for weekdays, weekend days, and all days combined. Regression analyses explored associations between physical activity outcomes, urban/rural residence, and a modified walkability index (dwelling density and intersection density); with interactions fitted for household equivalised income and season of data collection. Sensitivity analyses assessed variation in findings by socioeconomic factors and urbanicity.

**Results:**

Rural children spent an average of 14 min less sedentary (95% CI of difference: 2.23, 26.32) and 13 min more in light intensity activity (95% CI of difference, 2.81, 24.09) per day than those from urban settlements. No urban-rural differences were found for time spent in MVPA or in total levels of activity. Our walkability index was not associated with any outcome measure. We found no interactions with household equivalised income, but there were urban/rural differences in seasonal variation; urban children engaged in higher levels of MVPA in the spring months (difference: 10 mins, *p* = 0.06, n.s) and significantly lower levels in winter (difference: 8.7 mins, *p* = 0.036).

**Conclusions:**

Extrapolated across one-year, rural children would accumulate approximately 79 h (or just over 3 days) less sedentary time than urban children, replacing this for light intensity activity. With both outcomes having known implications for health, this finding is particularly important. Future work should prioritise exploring the patterns and context in which these differences occur to allow for more targeted intervention/policy strategies.

## Background

Physical inactivity is the fourth leading cause of death worldwide and is a key risk factor for a number of non-communicable diseases (NCDs) [1]. Sedentary behaviour has been identified as an independent risk factor for obesity and related cardiovascular and metabolic disease in young people [2]. Despite the well documented contribution of physical activity (PA) to reducing the risk of a number of NCDs [3], the world-wide prevalence of inactivity has remained stable since 2001 at approximately 27–28% [4, 5]. This is of particular concern for children and young people since inactive children tend to become inactive adults [6]. If we are to design better interventions to improve PA levels, we need a clearer understanding of the potential determinants, including whether these, or associated aspects, can be manipulated to lever change in behaviour.

The socio-ecological model (SEM) provides a framework for understanding the determinants of health behaviours such as PA [7]. Where other theories such as the Health Belief Model (HBM) or Theory of Planned Behaviour (TPB) [8, 9] tend to place emphasis on intrapersonal factors (e.g. individual biology, demographics, and psychological factors such as motivation), ecological models include a population focus, and make explicit reference to interdependencies between multiple spheres of influence, including intra and interpersonal factors, behavioural settings, the built, natural and socio-cultural environments, and policy factors [10]. The built and natural environment is thought to hold independent influence over children’s PA behaviours [10–12] and residence in urban and rural areas of the country has been associated with PA levels [13].

When described in the context of the SEM, urban and rural areas provide distinct settings through which the presence or absence of specific features and characteristics may influence opportunities for, and levels of, PA [14]. For example, urban and rural contexts may vary in terms of physical access to formal PA facilities, with those staying in urban areas living closer to population centres, which potentially contain a variety of different facilities [15]. Moreover, active travel opportunities vary as a result of distance to school, which is commonly shorter for urban children [16]. Inextricably linked is ‘walkability’, and urban areas have been considered more walkable, partly because of the greater availability of pavements/sidewalks [12]. In contrast, rural areas may afford better opportunities for unstructured outdoor activity/play and contact with nature [17, 18], yet rural children have also expressed concern regarding their limited access to leisure and recreational activities [19, 20].

With marked differences in how supportive the environment could be for PA and sedentary behaviour, there is potential for urban/rural differences in activity levels. However, the available international literature is equivocal over both magnitude and direction of any effect. Some papers suggest little or no differences [18, 21], whilst others have indicated that urban [11, 22] or rural children are more active [23, 24]. Mixed results are also evident when assessing sedentary behaviour [25–27]. A partial explanation might be the spatial distribution of deprivation in different countries. In the US for example, rural areas tend to be more deprived, whereas in the UK, the opposite is the case. The literature is dominated by studies from the US, Canada and Australia, with markedly few studies from the UK. Calls have been made to increase country specific studies to recognise the geographical heterogeneity of urban and rural built environments between countries. Doing so will allow for better comparison of findings and clarity over how much national context matters [18].

Previous studies have also recommended exploring factors that may mediate or moderate the link between environment and children’s PA/sedentary behaviours [12, 28, 29]. The natural environment, including climate, seasons, and weather influence PA levels [30]. Daylight length, temperature, and visibility have all been identified as significant positive predictors of children’s PA levels, with precipitation and wind speed found to be negative predictors [31]. In a UK context, these physical influences on being outdoors, or ease of getting to PA facilities, may be particularly important considerations but there is limited evidence about whether their impact is dependent on geographical location [13]. The impact of winter darkness may, for example, be much greater in a rural setting where street-lighting is sparse or less extensive given its close links to feelings of personal security and safety at night [32].

In addition to physical influences, socioeconomic factors may also moderate the relationship between PA/sedentary behaviour and urban-rural location. Lamb and colleagues [15] for instance, found an interaction between area level deprivation and urban rural residence when exploring access to PA facilities. Furthermore, Macdonald and colleagues [33] have recently shown that catchment areas around primary schools in more *deprived* areas of Scotland are more walkable (measured by a combination of path and road connectedness (i.e. intersection density), in addition to dwelling density), with their levels of walkability *decreasing* gradually as areas became more affluent. Given evidence suggesting that urban areas are also more walkable [12], the interacting effects of both urban/rural status and level of deprivation may have a strong influence of PA levels.

The primary aim of this paper was to explore urban and rural differences in the device-measured PA (total, light, and MVPA) and sedentary levels of young people across the whole of Scotland, UK. The secondary aims were to: i) evaluate the independent relationship of neighbourhood walkability on daily PA and sedentary time; and ii) explore the potential moderating relationship of season and individual or area level deprivation on urban-rural differences in PA and sedentary behaviours.

## Methods

Data came from the SPACES (Studying Physical Activity in Children’s Environments across Scotland) study, the aims and methods of which have been reported elsewhere [34]. Briefly, participants were recruited from Growing Up in Scotland (GUS); an on-going Scottish longitudinal cohort study that began in 2004 [35]. The original sample (*n* = 5217) was derived using the UK Child Benefit records and employed a cluster stratified sampling model using aggregated small geographical areas (Datazones, see definition below) as the primary sampling unit. From a possible 2402 children who had participated in the most recent GUS interview (aged 10/11 years old), 90% (*n* = 2162) of parents consented to be contacted by us, and were sent study information, registration documents, consent forms, and study devices (e.g. accelerometers) by post using the main parent/carer as primary contact. Data collection began in May 2015 and ran consecutively for 1 year, finishing in May 2016. Ethical approval was provided by the College of Social Sciences, University of Glasgow (CSS ref.: 400140067).

### PA measurement

Participants were asked to wear the validated [36, 37] ActiGraph GT3X+ accelerometer over eight consecutive days during waking hours. We considered days as valid if worn for 10 h on weekdays, and 8 h on weekend days [38]. This decision recognises the importance of having enough data to reliably represent daily activity yet adjusts for lower wear time during weekend days [39]. Children were asked to remove the accelerometer during sports or activities that may cause injury or harm, and when bathing or during other water-based activities. Following the measurement protocol set out by the International Physical Activity and Environment Network (IPEN), the following criteria were applied: i) a non-wear period was identified as 60 consecutive minutes of zero acceleration recorded by the device. Non-wear time periods were removed from further analyses; ii) children who provided at least 5 days including four weekdays and one weekend day were included in the analyses [40].

### PA data processing

Accelerometer data were processed using Actilife v6.11.9, a proprietary software from the ActiGraph manufacturer. The acceleration signal was extracted from the x-axis, digitised and stored as ‘counts’ – a unit-less representation of acceleration for that period. Raw data was processed into 10-s epochs.

The primary measure used to capture total physical activity was the participant’s counts per minute (cpm) - a measure of that integrates all movement recorded through the device over the duration of the device-wearing period (total counts recorded through the device divided by the length of time in minutes that the device was worn). These counts were then translated into time spent sedentary and in each intensity of PA by using an evidence-based threshold classification: sedentary (≤100 cpm); light (101–2295 cpm); and MVPA (≥2296 cpm) [41, 42].

### Urban and rural classification

To classify children according to their residence in urban or rural areas of Scotland with sufficient power for our analyses, we used the Scottish Government’s two-category classification system [43]. This defines urbanicity or rurality by the population size of a settlement (greater or less than 3000 people). Settlements are defined as a group of high density postcodes (i.e. more than 2.1 residential addresses per hectare, or population per hectare greater than five) whose combined population rounds to 500 people or more [44]. They are separated by low density postcodes.

To assess the sensitivity of our analyses to this choice, we also employed the Scottish Government’s six-category classification, which considers both population size of the settlement and remoteness/accessibility (based on drive time to the nearest settlement with a population of 10,000 people or more) [43]. We identified and compared children living in ‘Large Urban’ settlements with those living in ‘Remote Rural’ areas (Table 1). We also tested the population density of the data zone of residence as a continuous proxy for rurality. Data zones are small administratively defined neighbourhood areas of approximately 500–1000 household residents used in Scotland for statistical reporting [45], and the 2011 update was used in our analyses. Children were allocated to a classification or population density value based on their residential address.

**Table 1:** Classification of urban and rural settlements using a six-category or two-category system

Six-category classification	Two-category classification	Population size	Remoteness (drive time to ‘Other Urban’ settlement)
Large Urban	Urban	≥125,000	–
Other Urban	Urban	10,000 – 124,999	–
Accessible Small Town	Urban	3000 – 9999	< 30 min
Remote Small Town	Urban	3000 – 9999	> 30 min
Accessible Rural	Rural	< 3000	< 30 min
Remote Rural	Rural	< 3000	> 30 min

### Walkability

Following the work by Macdonald and colleagues [33], we adapted and applied a walkability index to the dataset to explore the relationship between the walkability score (WS) and daily physical activity/sedentary time, and to evaluate the independent relationship between urbanicity and PA/sedentary levels once controlling for our walkability measure. A full description of the index can be found elsewhere [33]. Briefly, we include dwelling density and street/path intersection density in a two-component index calculated at data zone level. Dwelling density was included as a measure of *proximity* and calculated from Scottish Neighbourhood Statistics [46] as the ratio of residential units to the land area using a count of number of dwellings, and land area in hectares, for each data zone (measured in 2016).

Street/path connectivity is characterised by ease of movement between places in the environment (e.g. between home and school, or home and friend’s house) [47]. A street network dataset and a path network dataset for Scotland (both relating to 2016) [48] were obtained from Ordnance Survey. Geographic Information System (GIS) mapping software (ArcMap v10) was used to combine the street network dataset (measured in 2016) with the path network via respective nodes, and for each data zone a measure of street/path connectivity was calculated using intersection density, i.e. the ratio of the number of true intersections (three or more legs) to the data zone area [49]. Z-scores were computed using IBM SPSS Statistics V.21 for both variables to standardise scores, and the following formula was used to calculate walkability score: WS = (2 × intersection z-scores) + (dwelling density z-scores) [50]. Street connectivity was weighted by two as previous work highlights the strong influence of this measure on walking [47]. All GIS work was conducted by Macdonald.

### Other built environment covariates

#### Distance to school

Distance from home to school may also influence daily PA in children due to its impact on active travel [51, 52]. The network distance (metres) was calculated from each child’s home location to their school using the gmapsdistance package [53] within R 3.2.0 in February 2018. The software calculated the shortest distance between these two precise geolocations using the Google Maps™ road and path network, for a walked journey.

#### Measurement of family socioeconomic position

We linked household income data from the most recent GUS survey (2014–2015) to each individual involved in the SPACES study. Household equivalised income was derived using the OECD (Organisation for Economic Co-operation and Development) modified equivalence scale [54] which adjusts household income to reflect the different resource needs of single adults, any additional adults in the household, and children in various age groups. Household income was rendered into quintiles to better model the known non-linear relationship between socioeconomic position and PA [45]. For sensitivity analysis we also used a measure of the data zone’s socio-economic deprivation. This was defined as the percentage of the data zone population (adults and their dependants) in receipt of UK social benefits (Income Support, Employment and Support Allowance, Job Seekers Allowance, Guaranteed Pension Credits, and Child and Working Tax Credits).

#### Measurement of season

Season was classified into a four-level categorical variable (Winter, Spring, Summer, Autumn) and reflected the data collection period when each participant wore the activity monitors. The associated start dates for seasonal classification were as follows: 20th March 2015 (Spring), June 21st 2015 (Summer), September 23nd (Autumn), 23rd December 2015 (Winter), 20th of March 2016 (Spring).

### Statistical analysis

Analyses were conducted using STATA v.14.2 (STATA Corporation, Texas, USA), and accounted for the clustered and stratified survey sample design of the GUS cohort [35]. Sampling weights were applied to allow for non-consent to contact, and non-consent and non-compliance of those invited to take part.

Linear regression models using Ordinary Least Squares (OLS) fit were conducted on continuous outcome variables (cpm, light, MVPA, and sedentary time) in two stages: Model 1 included the primary ‘predictor’ urban/rural status as a two-level factor variable, controlling for household equivalised income (quintile bands), sex, mean wear time per day, number of valid days, and season of measurement. Model 2 was as Model 1 but included the built environment characteristics ‘distance to school’ (metres), and data zone walkability z-score. These were followed by models that included interaction terms allowing the relationship between urban/rural location and the PA/sedentary outcomes to vary by household equivalised income and by season of measurement. The significance of interaction terms was assessed by adjusted (for survey design) Wald tests. To make the results of the interaction analyses easier to interpret, we estimated predicted values from the models using the Stata ‘margins’ command holding continuous variables at their mean and factor variables as if balanced. All models satisfied the required assumptions of OLS linear regression.

All analyses used complete case data and no imputation was carried out. In total, 774 children (417 girls, 357 boys; mean age 11.1 years) provided at least four weekdays of valid data and at least 1 day of valid weekend data, but 5% of these had some missing covariate data to leave 736 in the final analytical sample. There were no significant differences in any outcome measure (*p* > 0.05) between those included or excluded from the final sample, and missing covariates were not related to sex, living in urban or rural settlements, or measures of family socioeconomic position (p > 0.05).

## Results

### Sample weighting and descriptive statistics stratified by urban/rural classification

We compared our weighted sample with that of the GUS weighted Sweep 8 sample to examine representativeness (the GUS weighted sample is broadly representative of the population) (see Additional file 1). The weighting procedure was largely successful across all variables, with only minor differences compared to the entire GUS Sweep 8 sample. For instance, our weighted sample slightly under-represented those in lowest and highest income bands (<£3999 – £9999; > 50 k), those whose parents were married, those whose mothers were aged under 20 years old at the birth of their child, those with no educational qualifications in the household, and those who reside in ‘Large Urban’ areas of Scotland. The sample slightly over-represented those who were cohabiting or single, and those households with educational qualifications of ‘Higher Grades or equivalent’.

Table 2 presents the weighted sample characteristics stratified by urban/rural classification. Rural children lived significantly further away from school (1.6 km vs 3.5 km, urban and rural respectively) and wore their activity monitors for longer on weekends. Rural areas were also significantly less walkable as measured by our WS.

**Table 2:** Weighted covariate descriptive statistics by urban/rural dwelling classification

Demographic variable	Urban (*n* = 619)	Rural (*n* = 154)
Gender (% female)	54%	49%
Age (SD)	11.1 (0.3)	11.1 (0.3)
Household Equivalised Income (per annum)^a^		
Bottom Quintile (≤£13,450)	27%	18%
2nd (≥£13,451 < £22,827)	24%	23%
3rd (≥£22,827 < £29,375)	19%	24%
4th (≥£29,375 < £39,216)	14%	21%
Top Quintile (≥£39,216)	17%	14%
Season of measurement		
Winter	22%	24%
Spring	12%	11%
Summer	18%	21%
Autumn	48%	43%
Distance to school kilometres	1.5 (2.3)	3.1 (6.7)^‡^
Walkability density score	0.3 (2.3)	−2.5 (1.1)^‡^
Number of Valid Days (SD)	7.6 (1.0)	7.8 (1.0)
Weekdays	5.6 (0.9)	5.7 (0.9)
Weekend days	2.1 (0.7)	2.1 (0.7)
Mean Weartime (all days, SD)	778.4 (44.0)	777.8 (48.0)
Weekdays	800.5 (45.0)	794.0 (49.8)
Weekend days	712.6 (81.2)	730.4 (83.2)^*^
BMI UK categories		
Underweight	2%	2%
Healthy weight	64%	64%
Overweight	18%	15%
Obese	16%	19%

Significance testing conducted using adjusted Wald tests for continuous variables and chi-square for categorical

*SD* standard deviation

^*^p < 0.05

^‡^*p* < 0.01

^a^Missing covariate information: Urban *n* = 594; Rural *n* = 145

### Main findings

Following adjustment (Model 2 – fully adjusted model; see Additional file 3 for Models 1 and 2), no statistically significant differences in mean total activity (cpm) or MVPA per day for all days combined, or weekday and weekend days separately, were found between children living in urban or rural (two-category classification) settlements (Table 3). Rural children spent on average 13 min more per day in light intensity activity, and this difference was statistically significant for all days combined and separately for weekdays and weekend days. Urban children spent on average 14 min more per day sedentary, and this was statistically significant for all days combined and separately for weekdays.

**Table 3:** Adjusted means (95% CI) of outcome variables between urban and rural children

Outcome variable^a^	Urban (*n* = 591) mean (95% CI)	Rural (*n* = 145) mean (95% CI)	Diff mean (95% CI)
Mean Daily total activity, CPM	644.7	658.0	−13.23
	(622.9; 666.6)	(620.9; 695.1)	(− 53.84; 27.37)
Mean Weekday CPM	643.7	669.9	−26.25
	(623.3; 664.0)	(633.2; 706.6)	(− 68.55; 16.05)
Mean Weekend CPM	641.4	618.9	22.53
	(601.0; 681.9)	(556.9; 680.8)	(−38.17; 83.24)
Mean Daily Light Physical Activity, LPA (mins)	252.0	265.1	−13.08^*^
	(247.7; 256.3)	(255.8; 274.3)	(−23.05; −3.10)
Mean Weekday LPA	262.1	275.6	−13.45^*^
	(257.3; 266.9)	(265.6; 285.5)	(−24.09; − 2.81)
Mean Weekend LPA	223.1	235.9	−12.81^*^
	(217.3; 228.9)	(224.5; 247.3)	(−24.91; −0.72)
Mean Daily MVPA (mins)	72.3	73.5	−1.20
	(69.5; 75.0)	(68.7; 78.3)	(−6.55; 4.14)
Mean Weekday MVPA	74.8	77.6	−2.77
	(72.0; 77.7)	(72.6; 82.6)	(−8.32; 2.77)
Mean Weekend MVPA	64.8	61.0	3.79
	(60.8; 68.8)	(54.5; 67.6)	(−3.53; 11.11)
Mean Daily Sedentary time, ST (mins)	453.9	439.7	14.27^*^
	(447.9; 459.9)	(428.3; 451.0)	(2.23; 26.32)
Mean Weekday ST	462.1	445.9	16.22^*^
	(455.8; 468.4)	(433.8; 457.9)	(3.47; 28.98)
Mean Weekend ST	428.9	419.8	9.04
	(420.9; 436.9)	(406.2; 433.5)	(−4.92; 23.00)

*MVPA* Moderate to Vigorous Physical Activity, *LPA* Light Physical Activity, *ST* Sedentary Time

Due to rounding and decimal places, differences may not match subtractions; ^*^p < 0.05

^a^Marginal means, adjusted for household equivalised income, sex, body mass index (BMI), distance to school (metres), mean wear time per day, walkability z score, number of valid days, and season of measurement

Our sensitivity analyses, using population density and a six-category classification system to assess rural/urban status, showed no substantive differences to our main results (results not shown). Using the six-category classification, we also conducted post estimation tests contrasting those living in the most extreme urban (Large Urban, *n* = 265) and rural (Remote Rural, *n* = 53) settlements. Although similar patterns (compared to the two-category classification) emerged with reference to time spent sedentary and in light activity, no statistically significant differences were found (Additional file 2).

### Walkability

WS was significantly related to household income (*F* = 6.79, *p* < 0.001; less walkable as household income increased) and urban and rural status (β = 2.85, *p* < 0.001; urban = 0.3 vs. rural = − 2.5). The addition of WS to the PA/sedentary regression models made negligible improvement to the explained variance (see Additional file 3: Table S2 for baseline and fully adjusted models). As an independent variable, it did not significantly predict any PA/sedentary outcome (*p* > 0.3 across all variables).

### Moderation

The relationships between the PA or sedentary outcomes and urban/rural settlement (two-category classification) did not vary significantly by household income quintile. Sensitivity analyses using data zone level income deprivation did not reveal any substantive differences to this result. However, low levels of income deprivation in rural areas in Scotland resulted in large confidence intervals, making it difficult to reveal any meaningful effect.

**Fig. 1:**
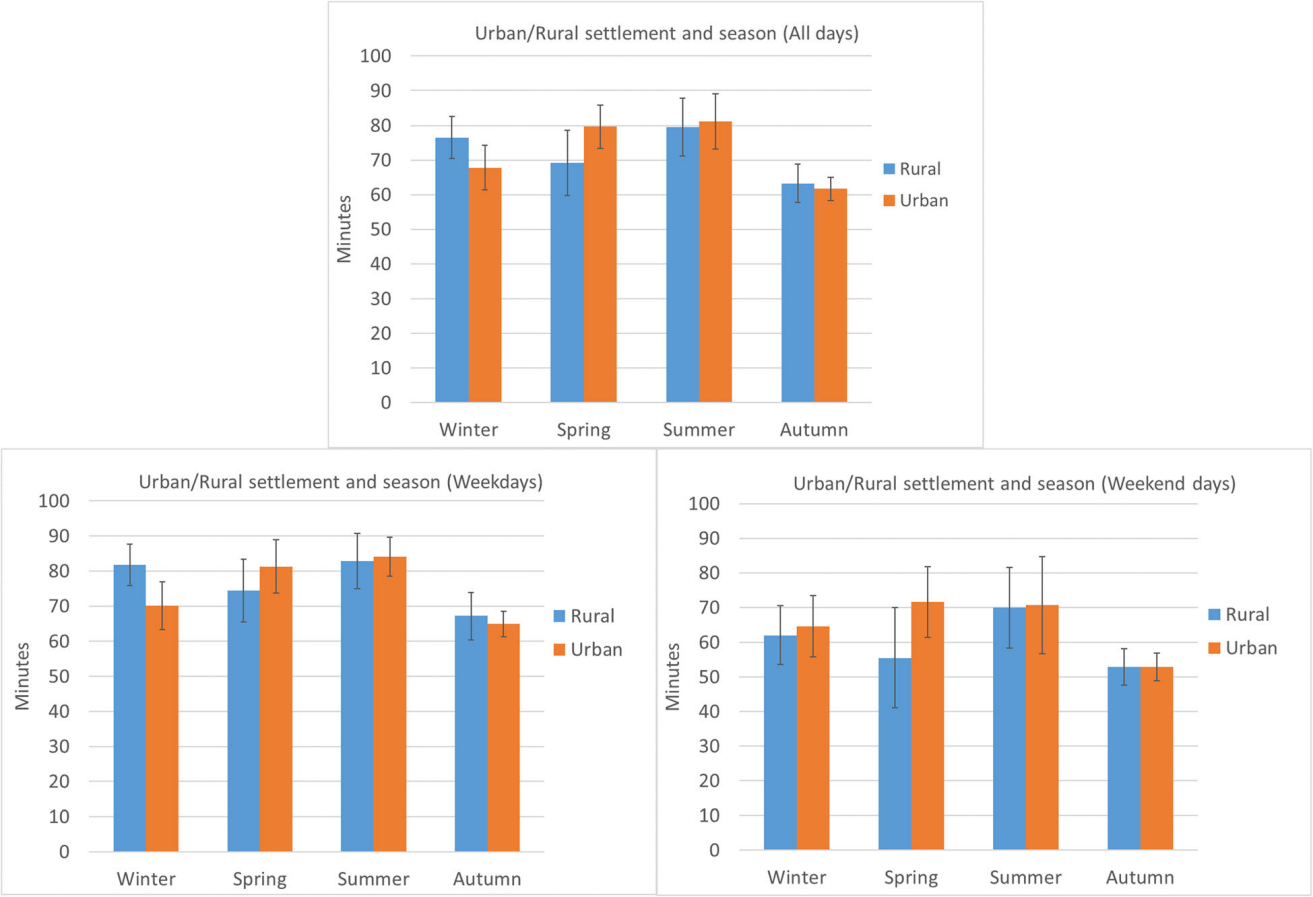
Adjusted marginal means of daily MVPA for urban and rural children by season (all days combined, weekdays, and weekend days)

There were however, statistically significant differences in the relationship between urban/rural location and time spent in MVPA, by season of measurement. These were found using all days combined, and weekdays only (Fig. 1). Levels of MVPA were highest (both urban and rural) in the summer months and lowest in the autumn months when analysing all days combined and weekdays. Urban children engaged in higher levels of MVPA per day (all days combined) in the spring months (difference: 10 mins, *p* = 0.06, n.s) but significantly lower levels in winter (difference: 8.7 mins, *p* = 0.036) than their rural counterparts. A similar pattern existed when we investigated weekdays only, however statistically significant differences were only evident between winter months (12 mins difference, *p* = 0.005). No statistically significant interaction was found for weekend days only.

## Discussion

We found no evidence of urban-rural differences in children’s daily MVPA or overall activity, in Scotland, UK. Although our WS varied between more and less deprived neighbourhoods, and between urban and rural areas, we did not find associations between WS and children’s daily physical activity. However, following model adjustments, our results did suggest that urban children spent approximately 14 min more sedentary per day than those from rural settlements, and this difference was greater during weekdays (16 min). This was explained by equivalent but reversed significant relationships in light intensity activity, where lower levels of sedentary time among rural children were displaced for higher levels of light activity (approximately 13 min difference across all outcomes levels). We found no evidence for moderation by household equivalised income, but seasonal dependence in urban/rural differences was observed for time spent in MVPA, where urban children engaged in significantly higher levels of MVPA in the spring months but lower levels in winter. Our results did not seem affected by choice of urban-rural indicator or measure of socioeconomic disadvantage.

There is a common vision of rural life as one of utopian splendour; idyllic places of peace [55], with rolling green hills, and nature at the doorstep [56]. With the potential environmental affordances that rural living can provide (e.g. wide, expansive fields for running), it may have been expected to translate to higher levels of MVPA. However, previous research has found little evidence of rural childhood automatically equating to closer engagement with nature, with participants reporting natural spaces being fenced off and/or inaccessible [56]. Children appear to both value and prefer to meet in outdoor spaces [17], yet this does not, based on our findings, translate to more MVPA. It does seem to translate to more light PA.

The majority of studies included in a recent narrative review tended to focus on MVPA or the meeting of PA guideline recommendations [21]; light PA and sedentary behaviour were explored far less despite the findings that light activity has been shown to be favourably associated with children’s cardiometabolic biomarkers (e.g. blood pressure, insulin resistance, high-density lipoprotein cholesterol) [57], and has an important impact on total daily energy expenditure [58]. Children in our study engaged in over 4 h of light activity per day, contributing approximately 30% of total average wear time (compared to 9% for MVPA). Rural children engaged in significantly more light activity than urban children on both weekdays and weekend days, amounting to approximately 13 min per day on average. To put this into perspective, across 1 year, this would constitute a difference of around 79 h, or just over 3 days. There is limited evidence about the dose reduction (of sedentary time) or increase (of light PA) required to affect health outcomes in children and young people. Nonetheless, in a recent systematic review by Carson and colleagues [2], the authors described a consistent gradient of worsening health by higher sedentary behaviours across 73 studies. Taken together, it is likely that the accumulated increase (light PA) and reduction (sedentary) over time will be positively related to health. It remains important to determine when, where, and why differences in light activity occur.

Our observed interaction with season is consistent with a recent large-scale UK analysis of the objectively measured levels of MVPA in 7 year old children which also found a season urban/rural interaction (*p* = 0.05) [59]. Similar to our findings, children from urban areas were most active in spring and summer months and least active in the autumn and winter months. In contrast to our findings, rural children were least active in the winter months, followed by autumn and then summer and spring. More research is required to explore seasonal variation in specific activity types (such as organised sport, winter/summer activities, or active travel behaviours) as a means of explaining the seasonal effects [59]. Informed by our current work [60], the next step is to integrate Global Positioning System (GPS) derived locations alongside novel Geographic Information Systems analytical techniques to capture mobility and environmental exposure. Doing so will lead to a greater understanding of the role of rurality and urbanicity in children’s physical activity, leading to more targeted intervention and policy decisions.

### Strengths, limitations, and future recommendations

This study used accelerometry data from a large-scale, country wide sample and as such provides much needed population level device-measured evidence of the effects of living in urban or rural areas on PA and sedentary levels of 10/11 year old children. Our results will be broadly representative and generalizable across the country, achieved through the statistical weighting of our analyses. Although largely successful, some under and over representation remained across a few socioeconomic variables but we do not believe this will have altered the interpretation of the findings.

The ability to incorporate seasons allowed us to explore the associations between periods of the year where weather conditions vary, and our analyses exploring urban/rural differences by socioeconomic factors was directly in response to the future recommendations of previous work in the field. Although a perceived strength, waist mounted devices do have their limitations and are typically poor at recording the acceleration associated with cycling or upper body dominant activities [12]. The devices were removed when in water and when engaging in contact sports, so we may have underestimated these activities and thus cannot speculate on the prevalence of either in our urban or rural participants. Additionally, whilst our chosen cut points to classify PA outcomes were evidence based, other published cut points are available [42], and their use may alter our results.

To maximise the power in our analyses, we used an urban/rural dichotomy based on *population size of a settlement* to classify the built environment exposure. This binary categorisation is recognised and used by the Scottish Government to identify issues and assist with policy making decisions in Scotland making the results particularly relevant and important. We strengthened our findings by re-running the analyses two further ways with little substantive differences in outcomes: i) we integrated ‘*remoteness*’ into our urbanicity measure by comparing children living in ‘Large Urban’ areas (high population) against those living in ‘Remote Rural’ areas (low population and greater than a 30 min drive time from an urban settlement); ii) we also used an area-based population density measure as a proxy of social and physical sparseness.

Our walkability measure used two components. Some other indices have also included other components, such as land-use mix, and net retail area [49]. Research using these has found associations with PA in adults [49, 61], however the role of such components in walkability indices for children is poorly understood. Built environment factors associated with adults’ PA may be less relevant to child populations [62]. Future analyses could include additional factors with greater relevance to children’s physical activity, such as traffic exposure [63] cul-de-sac density, private garden access, or public park, play- or sports-ground access [64].

Future research may wish to explore ‘remoteness’ in greater detail by moving beyond a 30 min drive time and capturing those children who stay in very remote areas (e.g. those living greater than a 60 min drive from an urban settlement), however, our study would have been underpowered to conduct these analyses with the current sample. Researchers may also consider alternative approaches to defining urban and rural status. This paper used a nationally recognised definition because of its direct applicability to decision making at policy level; however, there would be value in comparing this definition (and importantly, the findings) to a conceptually different one. For example, one could create an individualised index of urbanicity that combines multiple measures of the built environment (e.g. population density, number of homes, number of intersections, number of recreational facilities, area of farming/open area etc) based on standardised residential neighbourhoods (e.g. 1 km network buffer around the home). Combined with work that explores the qualitative meaning behind urban and rural living with regards to lifestyle and location [21], this may improve our understanding of urbanicity and its impact on PA levels and behaviours. Additionally, future work should explore the detailed individualised environmental context, exposure (e.g. to specific weather conditions if data is available), and land uses [65], underpinning the PA levels of urban and rural children across Scotland, and should also incorporate domain specific PA variables (e.g. active travel) to better reflect associations with certain ‘predictors’ (e.g. walkability and distance to school). Doing so will provide a rich account of when and where PA and sedentary time is accumulated, offering particular value for intervention development, planners, and policy makers.

## Conclusion

In conclusion, rural children spent significantly less time sedentary and more time in light activity than their urban counterparts. Light activity is seldom reported in the urban/rural literature, yet the findings from this study suggest that these differing environments may influence this specific component of PA. This study also provides support to a growing body of literature suggesting that season impacts on the levels of PA in children, and that this impact may manifest differently in urban and rural areas of the country. The results from this study will provide evidence for policy makers who are responsible for evaluating progress against national strategies aimed at combating physical inactivity. Although daily MVPA was identical in both urban and rural contexts, the interaction effect of urban/rural living with the natural seasons on levels of MVPA may influence future discussions on where and how national budgets should be spent. Future work that provides detailed context to the PA levels of those who stay in urban and rural areas should be prioritised to allow us to evaluate where and when PA is accumulated.

## Supplementary information

**Additional file 1.** Comparison of weighted sample to known national level SES/demographic distributions.

**Additional file 2.** Adjusted mean outcomes for all days combined (Top), weekdays (Middle), and weekend days (Bottom) comparing Large Urban vs. Remote Rural categories.

**Additional file 3.** Baseline and fully adjusted linear regression models exploring relationship between Urban/rural living and physical activity outcomes (all days combined, weekdays, and weekend days separately).

## Data Availability

We are committed to maximizing the use of SPACES study data to advance knowledge to improve young people’s health, and welcome proposals for collaborative projects and data sharing. Our data sharing policy follows that of the Medical Research Council and aims to balance making data as widely and freely available as possible while safeguarding the privacy of participants, protecting confidential data, and maintaining the reputation of the study. Access to raw data may be possible but will require consultation with partners at the Scottish Government. Please contact Prof Rich Mitchell (richard.mitchell@glasgow.ac.uk) for further information.

## Abbreviations

CMO: Chief Medical Officers
CPM: Counts per minute
GIS: Geographic Information System
GPS: Global Positioning System
GUS: Growing Up in Scotland
MVPA: Moderate to vigorous physical activity
NCD: Non-communicable disease
OECD: Organisation for Economic Co-operation and Development
PA: Physical activity
SPACES: Studying Physical Activity in Children’s Environments across Scotland
