# Supplementary figures and images for: The relationship between living in urban and rural areas of Scotland and children’s physical activity and sedentary levels: a country-wide cross-sectional analysis
Source: BMC Public Health. 2020 Mar 6;20:304. doi: 10.1186/s12889-020-8311-y (PMC7065337; doi:10.1186/s12889-020-8311-y)

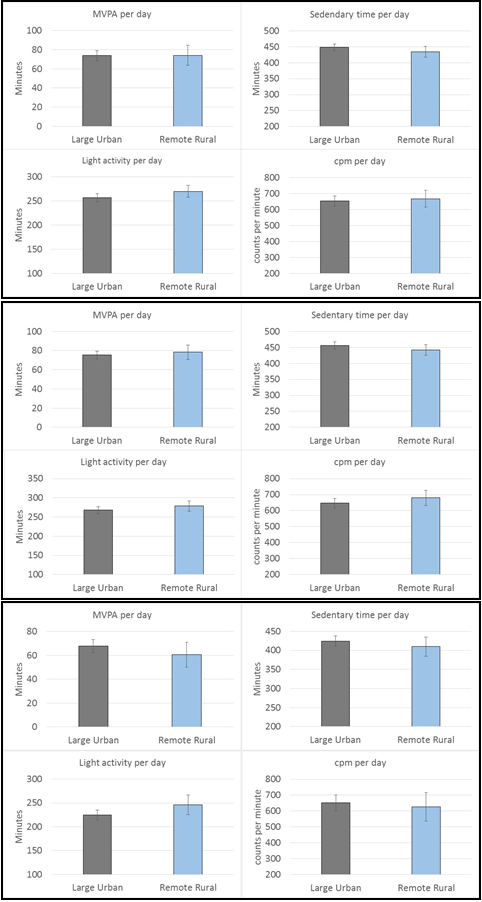

Supplement: Supplementary file 2 — Additional file 2. Adjusted mean outcomes for all days combined (Top), weekdays (Middle), and weekend days (Bottom) comparing Large Urban vs. Remote Rural categories. [file 12889_2020_8311_MOESM2_ESM.png]
