# Supplementary material for: The relationship between living in urban and rural areas of Scotland and children’s physical activity and sedentary levels: a country-wide cross-sectional analysis
Source: BMC Public Health. 2020 Mar 6;20:304. doi: 10.1186/s12889-020-8311-y (PMC7065337; doi:10.1186/s12889-020-8311-y)
Supplement: Supplementary file 3 — Additional file 3. Baseline and fully adjusted linear regression models exploring relationship between Urban/rural living and physical activity outcomes (all days combined, weekdays, and weekend days separately). [file 12889_2020_8311_MOESM3_ESM.docx]

| \| All days combined \| Mean MVPA \| \|  \| \| Mean Sed \| \|  \| \| Mean Light \| \|  \| \| Mean CPM \| \| \| --- \| --- \| --- \| --- \| --- \| --- \| --- \| --- \| --- \| --- \| --- \| --- \| --- \| --- \| --- \| \|  \| (1) \| (2) \| \|  \| (1) \| (2) \| \|  \| (1) \| (2) \| \|  \| (1) \| (2) \| \| Constant \| 60.14  (18.93; 101.35) \| 62.69  (22.17; 103.20) \| \|  \| -41.60  (-152.68; 69.48) \| -45.42  (-157.42; 66.59) \| \|  \| -18.63  (-107.67; 70.41) \| -17.36  (-106.41; 71.69) \| \|  \| 1086.70  (684.53; 1488.86) \| 1097.93  (699.98; 1495.88) \| \| Urban \| 0.90  (-3.57; 5.37) \| -1.20  (-6.55; 4.14) \| \|  \| 9.23  (-1.43; 19.89) \| 14.28*  (2.23; 26.32) \| \|  \| -10.13*  (-18.12; -2.15) \| -13.08*  (-23.05; -3.10) \| \|  \| -9.55  (-43.98; 24.87) \| -13.23  (-53.84; 27.37) \| \|  \|  \|  \| \|  \|  \|  \| \|  \|  \|  \| \|  \|  \|  \| \| Lowest Quintile \| - \| - \| \|  \| - \| - \| \|  \| - \| - \| \|  \| - \| - \| \| 2nd Quintile \| -2.37  (-10.64; 5.90) \| -2.60  (-10.83; 5.63) \| \|  \| 3.17  (-17.73; 24.07) \| 2.84  (-17.79; 23.48) \| \|  \| -0,81  (-16.59; 14.97) \| -0.25  (-15.81; 15.31) \| \|  \| -35.90  (-97.18; 25.39) \| -38.92  (-100.01; 22.18) \| \| 3rd Quintile \| -3.29  (-10.99; 4.4) \| -2.91  (-10.68; 4.86) \| \|  \| 8.62  (-11.12; 28.26) \| 7.36  (-12.35; 27.06) \| \|  \| -5.33  (-20.28; 9.61) \| -4.44  (-19.40; 10.51) \| \|  \| -36.54  (-95.81; 22.74) \| -36.99  (96.38; 22.40) \| \| 4th Quintile \| -2.19  (-9.85; 5.47) \| -1.77  (-9.46; 5.92) \| \|  \| 3.89  (-17.36; 25.14) \| 1.83  (-19.27; 22.94) \| \|  \| -1.71  (-18.13; 14.72) \| -0.07  (-16.38; 16.24) \| \|  \| -32.72  (-96.22; 30.78) \| -35.14  (-98.20; 27.92) \| \| Top Quintile \| -2.97  (-10.91; 4.97) \| -2.08  (-10.05; 5.90) \| \|  \| 7.71  (-12.97; 28.40) \| 5.53  (-15.03; 26.09) \| \|  \| -4.74  (-20.50; 11.03) \| -3.45  (-19.01; 12.12) \| \|  \| -40.06  (-103.63; 23.50) \| -38.63  (-101.36; 24.10) \| \|  \|  \|  \| \|  \|  \|  \| \|  \|  \|  \| \|  \|  \|  \| \| Female \| -8.98***  (-13.28; -4.69) \| -8.94***  (-13.29; -1.26) \| \|  \| 3.27  (-6.67; 13.21) \| 3.84  (-5.95; 13.64) \| \|  \| 5.71  (-1.79; 13.21) \| 5.09  (-2.24; 12.42) \| \|  \| -21.39  (-16.11; 126.06 \| -19.28  (-51.90; 13.34) \| \| Winter \| - \| - \| \|  \| - \| - \| \|  \| - \| - \| \|  \| - \|  \| \| Spring \| 7.87  (-0.31; 16.05) \| 8.08*  (0.13; 16.02) \| \|  \| 11.58  (-8.32; 31.49) \| 11.95  (-7.66; 31.56) \| \|  \| -19.45*  (-34.75; -4.16) \| -20.03**  (-34.98; -5.08) \| \|  \| 54.97  (-16.11; 126.06) \| 57.90  (-11.21; 127.01) \| \| Summer \| 11.77**  (2.97; 20.57) \| 11.12*  (2.45; 19.79) \| \|  \| 0.31  (-22.02; 22.65) \| 1.44  (-20.67; 23.55) \| \|  \| -12.09  (-28.18; 4.00) \| -12.56  (-28.56; 3.44) \| \|  \| 65.63  (-2.66; 133.91) \| 63.19  (-4.59; 130.98) \| \| Autumn \| -6.97*  (-13.43; -0.52) \| -7.59*  (-13.93; -1.26) \| \|  \| 35.77***  (17.43; 54.10) \| 36.58***  (18.27; 54.89) \| \|  \| -28.80***  (-42.63; -14.97) \| -28.99***  (-42.90; -15.09) \| \|  \| -67.97*  (-120.45; -15.49) \| -71.05**  (-122.12; -19.99) \| \|  \|  \|  \| \|  \|  \|  \| \|  \|  \|  \| \|  \|  \|  \| \| Weartime‡ \| 0.7**  (0.3; 1.2) \| 0.8***  (0.3; 1.2) \| \|  \| 5.5***  (4.3; 6.7) \| 5.4***  (4.2; 6.7) \| \|  \| 3.8***  (2.8; 4.8) \| 3.8***  (2.8; 4.8) \| \|  \| -1.3  (-5.9; 3.3) \| -1.1  (-5.6; 3.3) \| \| Valid Days \| -2.33*  (-4.46; -0.20) \| -2.35*  -(4.47; -0.23) \| \|  \| 1.16  (-3.79; 6.11) \| 1.18  (-3.80; 6.15) \| \|  \| 1.18  (-2.61; 4.97) \| 1.18  (-2.61; 4.97) \| \|  \| -15.09  (-32.81; 2.63) \| -15.19  (-32.53; 2.15) \| \| BMI \| -1.34**  (-2.24; -0.45) \| -1.44**  (-2.34; -0.54) \| \|  \| 1.78  (-0.49; 4.04) \| 1.90  (-0.37; 4.18) \| \|  \| -0.43  (-2.11; 1.25) \| -0.46  (-2.14; 1.23) \| \|  \| -10.36  (-16.94; -3.78) \| -10.87**  (-17.58; -4.16) \| \| Distance to School± \| - \| -0.39**  (-0.63; -0.16) \| \|  \| - \| 0.15  (-0.40; 0.70) \| \|  \| - \| 0.24  (-0.22; 0.70) \| \|  \| - \| -3.03***  (-4.62; -1.44) \| \| WS \| - \| 0.34  (-0.87; 1.55) \| \|  \| - \| -1.67  (-3.72; 0.39) \| \|  \| - \| 1.38  (-0.30; 2.95) \| \|  \| - \| -1.95  (-9.96; 6.07) \| \| Observations \| 731 \| 731 \| \|  \| 731 \| 731 \| \|  \| 731 \| 731 \| \|  \| 731 \| 731 \| \| Population size \| 735.5 \| 735.5 \| \|  \| 735.5 \| 735.5 \| \|  \| 735.5 \| 735.5 \| \|  \| 735.5 \| 735.5 \| \| F Statistic \| 7.86*** \| 6.68*** \| \|  \| 9.52*** \| 8.02*** \| \|  \| 8.34*** \| 9.51*** \| \|  \| 5.11*** \| 4.63*** \| \| Population R^2^ \| 0.172 \| 0.183 \| \|  \| 0.25 \| 0.25 \| \|  \| 0.21 \| 0.21 \| \|  \| 0.13 \| 0.14 \| |
| --- | --- | --- | --- | --- | --- | --- | --- | --- | --- | --- | --- | --- | --- | --- | --- | --- | --- | --- | --- | --- | --- | --- | --- | --- | --- | --- | --- | --- | --- | --- | --- | --- | --- | --- | --- | --- | --- | --- | --- | --- | --- | --- | --- | --- | --- | --- | --- | --- | --- | --- | --- | --- | --- | --- | --- | --- | --- | --- | --- | --- | --- | --- | --- | --- | --- | --- | --- | --- | --- | --- | --- | --- | --- | --- | --- | --- | --- | --- | --- | --- | --- | --- | --- | --- | --- | --- | --- | --- | --- | --- | --- | --- | --- | --- | --- | --- | --- | --- | --- | --- | --- | --- | --- | --- | --- | --- | --- | --- | --- | --- | --- | --- | --- | --- | --- | --- | --- | --- | --- | --- | --- | --- | --- | --- | --- | --- | --- | --- | --- | --- | --- | --- | --- | --- | --- | --- | --- | --- | --- | --- | --- | --- | --- | --- | --- | --- | --- | --- | --- | --- | --- | --- | --- | --- | --- | --- | --- | --- | --- | --- | --- | --- | --- | --- | --- | --- | --- | --- | --- | --- | --- | --- | --- | --- | --- | --- | --- | --- | --- | --- | --- | --- | --- | --- | --- | --- | --- | --- | --- | --- | --- | --- | --- | --- | --- | --- | --- | --- | --- | --- | --- | --- | --- | --- | --- | --- | --- | --- | --- | --- | --- | --- | --- | --- | --- | --- | --- | --- | --- | --- | --- | --- | --- | --- | --- | --- | --- | --- | --- | --- | --- | --- | --- | --- | --- | --- | --- | --- | --- | --- | --- | --- | --- | --- | --- | --- | --- | --- | --- | --- | --- | --- | --- | --- | --- | --- | --- | --- | --- | --- | --- | --- | --- | --- | --- | --- | --- | --- | --- | --- | --- | --- | --- | --- | --- | --- | --- | --- | --- | --- | --- | --- | --- | --- | --- | --- | --- | --- | --- | --- | --- | --- | --- | --- | --- | --- | --- | --- | --- | --- | --- | --- | --- | --- | --- | --- | --- | --- | --- | --- | --- | --- | --- | --- | --- | --- | --- | --- | --- | --- | --- | --- | --- | --- | --- | --- | --- | --- | --- | --- | --- | --- | --- | --- | --- | --- | --- | --- | --- | --- | --- | --- | --- | --- | --- | --- | --- | --- | --- | --- | --- | --- | --- | --- | --- | --- | --- | --- | --- | --- | --- | --- | --- | --- | --- | --- | --- | --- | --- | --- | --- | --- | --- | --- | --- | --- | --- | --- | --- | --- | --- | --- | --- | --- | --- | --- | --- | --- | --- | --- |

± Coefficient scaled to represent the change in the outcome for every 500-metre increase in the distance children live from school

‡ Coefficient scaled to represent the change in the outcome for every 10-minute increase in weartime

*p<0.05; **p<0.01; ***p<0.001

(1) Model 1: Urban/Rural classification, Household Equivalised Income (Base level = lowest quintile)), Sex, Season of measurement (Base level = Winter), mean weekday weartime, Number of valid week days, BMI

(2) Model 2: As Model 1 with addition of Distance to School and Walkability Score (WS)

| Weekdays | Mean MVPA | |  | | Mean Sed | |  | | Mean Light | |  | | Mean CPM | |
| --- | --- | --- | --- | --- | --- | --- | --- | --- | --- | --- | --- | --- | --- | --- |
|  | (1) | (2) | |  | (1) | (2) | |  | (1) | (2) | |  | (1) | (2) |
| Constant | 28.59  (-8.13; 65.32) | 31.54  (-4.59; 67.67) | |  | -35.98  (-148.42; 76.47) | -41.54  (-154.89; 71.81) | |  | 7.27  (-85.72; 100.25) | 9.89  (-83.70; 103.49) | |  | 776.60  (509.61; 1043.58) | 787.03  (523.18; 1050.87) |
| Urban | 0.00  (-4.64; 4.64) | -2.78  (-8.32; 2.77) | |  | 10.44  (-0.74; 21.62) | 16.22*  (3.47; 28.98) | |  | -10.45*  (-19.20; -1.69) | -13.45*  (-24.09; -2.81) | |  | -17.77  (-53.70; 18.16) | -26.25  (-68.55; 16.05) |
|  |  |  | |  |  |  | |  |  |  | |  |  |  |
| Lowest Quintile | - | - | |  | - | - | |  | - | - | |  | - | - |
| 2nd Quintile | -1.76  (-10.09; 6.57) | -1.96  (-10.23; 6.32) | |  | 5.97  (-16.19; 28.12) | 5.62  (-16.25; 27.49) | |  | -4.22  (-22.06; 13.62) | -3.67  (-21.31; 13.97) | |  | -24.06  (-79.82; 31.70) | -26.62  (-82.29; 29.05) |
| 3rd Quintile | -2.68  (-10.76; 5.39) | -2.15  (-10.29; 6.00) | |  | 10.31  (-10.77; 31.38) | 8.86  (-12.31; 30.02) | |  | -7.63  (-24.07; 8.82) | -6.71  (-23.26; 9.84) | |  | -23.77  (-80.60; 33.06) | -22.98  (-80.50; 34.53) |
| 4th Quintile | -1.58  (-8.95; 5.78) | -0.92  (-8.44; 6.60) | |  | 7.60  (-13.79; 28.99) | 5.28  (-16.12; 26.67) | |  | -6.02  (-23.57; 11.53) | -4.37  (-21.91; 13.17) | |  | -19.63  (-72.62; 33.35) | -20.02  (-74.49; 34.45) |
| Top Quintile | -2.87  (-11.16; 5.41) | -1.71  (-10.07; 6.65) | |  | 9.92  (-11.58; 31.42) | 7.41  (-14.09; 28.91) | |  | -7.05  (-23.88; 9.79) | -5.70  (-22.44; 11.04) | |  | -29.20  (-87.84; 29.45) | -25.87  (-84.77; 33.03) |
|  |  |  | |  |  |  | |  |  |  | |  |  |  |
| Female | -10.34***  (-14.65; -6.02) | -10.37***  (-14.75; -5.99) | |  | 6.95  (-3.55; 17.45) | 7.58  (-2.82; 17.98) | |  | 3.38  (-5.01; 11.76) | 2.78  (-41.68; -4.51) | |  | -41.25  (-70.63; -11.87) | -39.90  (-69.64; -10.15) |
| Winter | - | - | |  | - |  | |  |  |  | |  |  |  |
| Spring | 7.39  (-1.88; 16.65) | 7.58  (-1.40; 16.56) | |  | 15.16  (-9.44; 49.76) | 15.51  (-8.73; 39.75) | |  | -22.55*  (-41.38; -3.71) | -23.09*  (-41.68; -4.51) | |  | 24.72  (-40.94; 90.38) | 27.27  (-37.12; 91.65) |
| Summer | 12.04**  (4.02; 20.06) | 11.24**  (3.44; 19.03) | |  | 3.32  (-18.98; 25.61) | 4.61  (-17.52; 26.74) | |  | -15.36  (-32.97; 2.26) | -15.85  (-33.41; 1.72) | |  | 58.57  (-1.57; 118.70) | 55.13  (-4.27; 114.53) |
| Autumn | -6.34  (13.40; 0.71) | -7.09*  (-13.98; -0.20) | |  | 40.13***  (20.43; 59.83) | 41.10***  (21.38; 60.82) | |  | -33.80***  (-49.16; 18.43) | -34.02***  (-49.52; -18.51) | |  | -69.92  (-122.88; -16.97) | -73.72  (-125.56; -21.88) |
|  |  |  | |  |  |  | |  |  |  | |  |  |  |
| Weekday Weartime‡ | 1.00***  (0.6; 1.5) | 1.10***  (0.6; 1.5) | |  | 5.2***  (3.9; 6.5) | 5.1***  (3.8; 6.5) | |  | 3.8***  (2.7; 4.9) | 3.8***  (2.7; 4.9) | |  | 1.5  (-1.5; 4.4) | 1.7  (-1.3; 4.6) |
| Weekday Valid Days | -1.74  (-3.98; 0.50) | -1.63  (-3.80; 0.53) | |  | 1.95  (-3.42; 7.32) | 1.96  (-3.47; 7.40) | |  | -0.21  (-5.02; 4.60) | -0.33  (-5.17; 4.52) | |  | -6.43  (-24.51; 11.65) | -5.51**  (-23.02; 12.01) |
| BMI | -1.23**  (-2.11; -0.35) | -1.35**  (-2.23; -0.47) | |  | 2.04  (-0.31; 4.39) | 2.19  (-0.18; 4.56) | |  | -0.81  (-2.64; 1.02) | -0.84  (-2.69; 1.01) | |  | -8.78  (-15.02; -2.54) | -9.41**  (-15.75; -3.08) |
| Distance to School± | - | -0.43**  (-0.70; -0.16) | |  | - | 0.21  (-0.39; 0.82) | |  | - | 0.21  (-0.29; 0.72) | |  | - | -3.04***  (-4.73; -1.36) |
| WS | - | 0.54  (-0.69; 1.78) | |  | - | -1.86  (-4.07; 0.35) | |  | - | 1.31  (-0.46; 3.09) | |  | - | -0.20  (-8.21; 7.81) |
| Observations | 731 | 731 | |  | 731 | 731 | |  | 731 | 731 | |  | 731 | 731 |
| Population size | 735.5 | 735.5 | |  | 735.5 | 735.5 | |  | 735.5 | 735.5 | |  | 735.5 | 735.5 |
| F Statistic | 9.75*** | 8.32*** | |  | 13.96*** | 11.70*** | |  | 6.22*** | 6.77*** | |  | 6.65*** | 5.96 |
| Population R^2^ | 0.18 | 0.19 | |  | 0.24 | 0.24 | |  | 0.20 | 0.20 | |  | 0.12 | 0.13 |

± Coefficient scaled to represent the change in the outcome for every 500-metre increase in the distance children live from school

‡ Coefficient scaled to represent the change in the outcome for every 10-minute increase in weartime

*p<0.05; **p<0.01; ***p<0.001

(1) Model 1: Urban/Rural classification, Household Equivalised Income (Base level = lowest quintile)), Sex, Season of measurement (Base level = Winter), mean weekday weartime, Number of valid week days, BMI

(2) Model 2: As Model 1 with addition of Distance to School and Walkability Score (WS)

| **Weekend** | Mean MVPA | |  | | Mean Sed | |  | | Mean Light | |  | | Mean CPM | |
| --- | --- | --- | --- | --- | --- | --- | --- | --- | --- | --- | --- | --- | --- | --- |
|  | (1) | (2) | |  | (1) | (2) | |  | (1) | (2) | |  | (1) | (2) |
| Constant | 69.77  (24.71; 114.83) | 70.74  (24.75; 116.73) | |  | -92.16  (-194.87; 10.54) | -92.88  (-195.18; 9.41) | |  | 22.36  (-46.04; 90.77) | 22.12  (-45.44; 89.68) | |  | 1208.40  (734.75; 1682.05) | 1216.70  (733.80; 1699.61) |
| Urban | 3.34  (-2.84; 9.52) | 3.79  (-3.53; 11.11) | |  | 7.52  (-5.54; 20.59) | 9.04  (-4.92; 23.00) | |  | -10.85*  (-21.06; -0.65) | -12.82*  (-24.91; -0.72) | |  | 11.17  (-42.18; 64.53) | 22.53  (-38.17; 83.24) |
|  |  |  | |  |  |  | |  |  |  | |  |  |  |
| Lowest Quintile | - | - | |  | - | - | |  | - | - | |  | - | - |
| 2nd Quintile | -2.34  (-15.47; 10.79) | -2.63  (-15.70; 10.44) | |  | -3.48  (-29.60; 22.63) | -3.68  (-29.74; 22.37) | |  | 5.82  (-11.19; 22.83) | 6.31  (-10.46; 23.07) | |  | -55.88  (-177.75; 66.00) | -60.04  (-181.76; 61.69) |
| 3rd Quintile | -3.54  (-13.71; 6.63) | -3.75  (-14.12; 6.52) | |  | 1.50  (-19.16; 22.16) | 1.09  (-19.87; 22.05) | |  | 2.04  (-11.56; 15.64) | 2.66  (-11.11; 16.43) | |  | -57.02  (-157.95; 43.90) | -61.11  (-162.04; 39.80) |
| 4th Quintile | -4.17  (-15.57; 7.24) | -4.65  (-15.75; 6.46) | |  | -3.31  (-27.04; 20.41) | -4.06  (-27.92; 19.81) | |  | 7.48  (-8.21; 23.17) | 8.70  (-7.30; 24.71) | |  | -68.38  (-181.98; 45.22) | -76.95  (-187.94; 34.04) |
| Top Quintile | -4.19  (-15.16; 6.78) | -4.39  (-15.13; 6.36) | |  | 2.79  (-19.07; 24.64) | 2.13  (-1974; 24.00) | |  | 1.40  (-13.65; 16.45) | 2.25  (-12.96; 17.47) | |  | -76.88  (-184.55; 30.79) | -81.83  (-186.85; 23.18) |
|  |  |  | |  |  |  | |  |  |  | |  |  |  |
| Female | -5.92  (-12.54; 0.71) | -5.69  (-12.42; 1.05) | |  | -2.28  (-15.67; 11.11) | -2.04  (-15.48; 11.40) | |  | 8.19  (-1.00; 17.39) | 7.72  (-1.47; 16.92) | |  | 19.32  (-42,18; 80.82) | 22.96  (-38.73; 84.65) |
| Winter | - | - | |  | - | - | |  | - | - | |  | - | - |
| Spring | 4.91  (-6.92; 16.73) | 5.21  (-633; 16.75) | |  | 5.68  (-16.24; 27.59) | 5.92  (-16.12; 27.97) | |  | 10.58  (-26.15; 4.98) | -11.14  (-26.53; 4.25) | |  | 94.88  (-56.10; 245.86) | 99.96  (-38.73; 84.64) |
| Summer | 7.01  (-6.35; 20.37) | 7.03  (-6.10; 20.16) | |  | -8.59  (-36.00; 18.82) | -8.25  (-35.26; 18.75) | |  | 1.57  (-15.78; 18.92) | 1.22  (-15.97; 18.42) | |  | 52.07  (-55.37; 159.51) | 53.58  (-52.92; 160.09) |
| Autumn | -10.85**  (-18.64; -3.05) | -10.94**  (-18.69; -3.20) | |  | 23.54*  (2.40; 44.68) | 23.72*  (2.69; 44.76) | |  | -12.69  (-28.81; 3.42) | -12.79  (-28.88; 3.29) | |  | -79.88*  (-150.00; -9.76) | -80.17*  (-149.10; -11.24) |
|  |  |  | |  |  |  | |  |  |  | |  |  |  |
| Weekend Weartime‡ | 0.46  (-0.05; 0.96) | 0.46  (-0.04; 0.95) | |  | 6.76***  (5.73; 7.79) | 6.75***  (5.71; 7.79) | |  | 2.79***  (2.13; 3.43) | 2.80***  (2.13; 3.46) | |  | -3.58  (-9.42; 2.26) | -3.61  (-9.31; 2.08) |
| Weekend Valid Days | -1.31  (-6.59; 3.96) | -1.44  (-6.72; 3.84) | |  | -1.53  (12.60; 9.53) | -1.57  (-12.76; 9.62) | |  | 2.85  (-4.31; 10.01) | 3.01  (-4.25; 10.28) | |  | -1.92  (-47.38; 43.54) | -3.54  (-48.81; 41,73) |
| BMI | -1.81**  (-2.98; -0.65) | -1.83*  (-3.03; -0.64) | |  | 1.57  (-0.97; 4.11) | 1.59  (-0.96; 4.14) | |  | 0.24  (-1.61; 2.10) | 0.24  (-1.59; 2.08) | |  | -16.23***  (-25.68; -6.78) | -16.39***  (-26.17; -6.60) |
| Distance to School± | - | -0.20  (-0.45; 0.04) | |  | - | -0.05  (-0.68; 0.58) | |  | - | 0.26  (-0.30; 0.81) | |  | - | -2.59*  (-5.14; -0.04) |
| WS | - | -0.38  (-1.95; 1.18) | |  | - | -0.61  (-3.33; 2.10) | |  | - | 1.00  (-1.03; 3.02) | |  | - | -6.94  (-18.65; 4.78) |
| Observations | 731 | 731 | |  | 731 | 731 | |  | 731 | 731 | |  | 731 | 731 |
| Population size | 735.5 | 735.5 | |  | 735.5 | 735.5 | |  | 735.5 | 735.5 | |  | 735.5 | 735.5 |
| F Statistic | 3.96*** | 3.74*** | |  | 16.21*** | 14.31*** | |  | 9.68*** | 8.95*** | |  | 2.19* | 2.40* |
| Population R^2^ | 0.97 | 0.10 | |  | 0.41 | 0.41 | |  | 0.21 | 0.21 | |  | 0.08 | 0.09 |

± Coefficient scaled to represent the change in the outcome for every 500-metre increase in the distance children live from school

‡ Coefficient scaled to represent the change in the outcome for every 10-minute increase in weartime

*p<0.05; **p<0.01; ***p<0.001

(1) Model 1: Urban/Rural classification, Household Equivalised Income (Base level = lowest quintile)), Sex, Season of measurement (Base level = Winter), mean weekday weartime, Number of valid week days, BMI

(2) Model 2: As Model 1 with addition of Distance to School and Walkability Score (WS)
